# *Urtica dioica* L. inhibits proliferation and enhances cisplatin cytotoxicity in NSCLC cells *via* Endoplasmic Reticulum-stress mediated apoptosis

**DOI:** 10.1038/s41598-019-41372-1

**Published:** 2019-03-21

**Authors:** Brigida D’Abrosca, Vincenza Ciaramella, Vittoria Graziani, Federica Papaccio, Carminia Maria Della Corte, Nicoletta Potenza, Antonio Fiorentino, Fortunato Ciardiello, Floriana Morgillo

**Affiliations:** 1 Dipartimento di Scienze e Tecnologie Ambientali Biologiche e Farmaceutiche –DiSTABiF, Università degli Studi della Campania “Luigi Vanvitelli”, via Vivaldi 43, I-81100 Caserta, Italy; 2 Dipartimento di Medicina di Precisione, Università degli Studi della Campania “Luigi Vanvitelli” - Via Pansini, 5, 80131, Napoli, Italy

## Abstract

Non-small cell lung cancer (NSCLC) is the most common type of lung cancer and the ineffectiveness of the current therapies seriously limits the survival rate of NSCLC patients. In the search for new antitumor agents, nature has played a pivotal role providing a variety of molecules, which are likely to exert selective anti-tumour properties. Herein, we investigated the antiproliferative potential of *Urtica dioica* L. extract (UD) against NSCLC cell models with low sensitivity to cisplatin, a cytotoxic agent largely employed to cure NSCLCs. UD inhibited cell proliferation in the selected cells, while no toxic effects were observed in normal lung cells. Furthermore, the co-treatment of UD and cisplatin notably sensitised NSCLC cells to cisplatin. Mechanistically, we discovered that UD-promoted endoplasmic reticulum (ER) stress via activation of the growth arrest and DNA damage-inducible gene 153 (GADD153) triggering apoptosis. We also performed an extensive NMR analysis of UD, identifying rutin and oxylipins as the main secondary metabolites present in the mixture. Additionally, we discovered that an oxylipins’ enriched fraction contributes to the antiproliferative activity of the plant extract. In the future, this study may provide new chemical scaffolds for the design of anti-cancer agents that target NSCLCs with low sensitivity to cisplatinum.

## Introduction

Non-small cell lung cancer (NSCLC) is the most common type of lung cancer and the major cause of cancer-related deaths worldwide^1^. In this pathology, symptoms commonly appear at a later stage, consequently, at the time of diagnosis, a large part of patients is at an advanced stage of the disease. Until a few years ago, the only therapeutic option for advanced NSCLC patients was represented by cisplatinum-based combination therapies that yielded a limited outcome improvement with an average overall survival (OS) of <12 months and a 5-year survival rate of <1%. Although cisplatin has been considered one of the most potent anti-cancer agents for lung cancers as well as diverse solid tumours, clinical evidence pointed out that the occurrence of cisplatin resistance clearly limited the benefits of this drug^2^. The molecular mechanism underlying cisplatin resistance is extremely variegate; nevertheless, it is known that many of them have defects in their apopototic mechanism^3^. To date, molecular target agents have been introduced in clinical studies to inhibit specific mutated oncoproteins in molecularly-selected NCSLCs. These primarily included tyrosine kinase inhibitors (TKI) against the epithelial growth receptor (EGFR), such as gefitinib and erlotinib, which successfully counteract tumour progression in NSCLC patients harbouring genetic alterations of EGFR^4^. However, EGFR mutations occur in the 10–26% of NSCLC patients and the majority of them are still treated with standard chemotherapies, mostly represented by cisplatin^5^. Therefore, the development of new therapeutic strategies as well as adjuvant agents able to improve the sensitivity of NCSLC patients to cisplatin, remains of paramount importance.

Although the new technologies of combinatorial chemistry provide a wide range of new and synthetic anticancer drugs, natural products produced by medicinal plants have been the pillar of cancer chemotherapy for many years^6,7^. The evolutionary process led plants to synthesise secondary metabolites in order to respond to a plethora of abiotic and biotic stress. Nature designed these molecules with a well-defined three-dimensional structure in order to interact specifically with biological targets of interest. Interestingly, the structure of the biological targets (*e.g*. protein-binding size) of natural products are often highly conserved among human proteins^8^, and this makes plants an ideal source of molecules useful to develop new chemotherapeutic agents that counteract tumour targets in an extremely selective way^9^.

Preliminary data from our group along with previous works led us to select *Urtica dioica* L. as a potential source of new agents against NSCLC. *Urtica dioica*, also known as the stinging nettle, is a perennial herbaceous plant growing in temperate and tropical wasteland areas worldwide^10^. Since ancient times, it has been used as food, paint, in fibres, manure and cosmetics^11,12^. Moreover, it was widely used as a medicinal herb for a long time, but only recently has it been examined for its anti-oxidant, anti-microbial, anti-ulcer, analgesic and anti-cancer properties^13–19^. Previous reports highlighted the anti-tumour activity of *U. dioica* in different human malignancies, such as breast^17^ and prostate^15^ cancers. Yet, to our knowledge, no previous works have investigated the potential anti-cancer activity of UD in NSCLC.

Thus, this study aimed to understand whether the *U. dioica* extract (UD) exerts a selective anti-proliferative activity in NSCLC cell models with a low sensitivity to cisplatin, and if so, to elucidate the mechanism by which this activity occurs. Furthermore, the current study also intends to identify the main secondary metabolites of the plant mixture and to discover whether these contribute to the putative anti-proliferative effect of the plant extract.

## Results

### Selective cytotoxicity of *Urtica dioica* extract in human lung cancer cells

**Figure 1:**
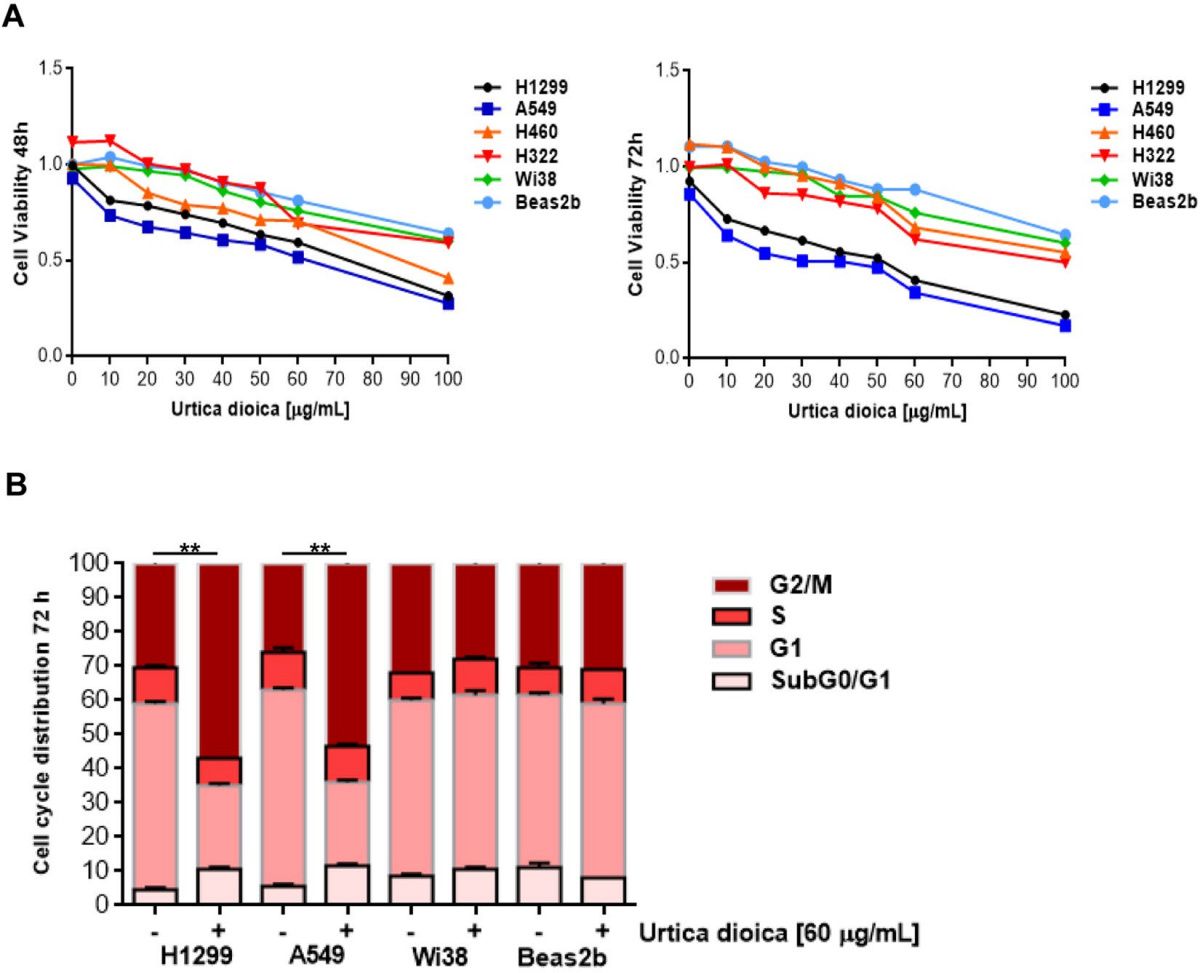
Effects of *Urtica dioica* extract on NSCLC (H1299, A549, H460 and H322) cell lines, normal bronchial epithelial (Beas2b) cells and human fibroblasts (Wi38). (**A**) *Urtica dioica* extract treatment was performed at the indicated doses for 48 and 72 h. Cell proliferation was measured with the MTT assay, as described in Materials and Methods, in human NSCLC cell lines and in normal bronchial epithelial cells and human fibroblasts. The results are the average ± sd of three independent experiments, each done in triplicate. All the used doses are statistically significant as determined by the Student-t test (**P ≤ 0.01). For sake of simplicity asterisks (**) are not reported in the graph. (**B**) Cell cycle distribution of H1299, A549, Beas2B and Wi38 cells after treatment with *Urtica dioica* extract for 72 h. Each experiment was done in duplicate ± sd. Statistically significant data are evidenced with asterisks (**P ≤ 0.01).

The cytotoxic activity of the UD extract was assessed on human NSCLC H460, H1299, A549 and H322 cell lines, which were previously selected by our group as EGFR wild-type cell models with a low sensitivity to cisplatin-based therapies^4^. Plant extract cytotoxicity was evaluated through MTT assay exploring a broad spectrum of doses in two time periods (48 and 72 h). Results from these experiments proved that UD decreased NSCLC cell proliferation in a time and dose-dependent manner (Fig. 1A). The studied cell lines showed a diverse sensitivity to the treatment, since the plant extract exerted a two-fold higher activity in H1299 and A549 (IC_50_ values: 52.333 ± 0.003 and 47.466 ± 0.003 µg/mL, for H1299 and A549 respectively) compared with H460 and H322 (IC_50_ values: 84.333 ± 0.002 and 78.333 ± 0.002 µg/mL for H460 and H322 respectively). H1299 and A549 are particularly refractory to cisplatin treatment^4^, thus these were selected for further investigations aimed at figuring out the underlying mechanism(s) by which nettle induces cell death. In addition to this, it was investigated whether the UD extract extended its cytotoxic effect on normal lung cells and, for this purpose, plant treatment was also performed on Beas2B and Wi38, normal bronchial epithelial and lung fibroblast cells, respectively. Interestingly, cytotoxicity was only appreciable at higher doses, suggesting that UD preferentially inhibited the growth of malignant lung cancer cells A549 and H1299 (Fig. 1A).

### Apoptosis induction by *Urtica dioica* extract in human lung cancer cells

In an attempt to understand how nettle impairs cell growth, firstly, the effect of the plant extract on the cell cycle by using a flow cytometer was determined. After treating cells with 60 μg/mL of UD for 3 days, H1299 and A549 accumulated in the G2/M phase; conversely, no detectable changes were noted in Beas2B and Wi38 (Fig. 1B).

**Figure 2:**
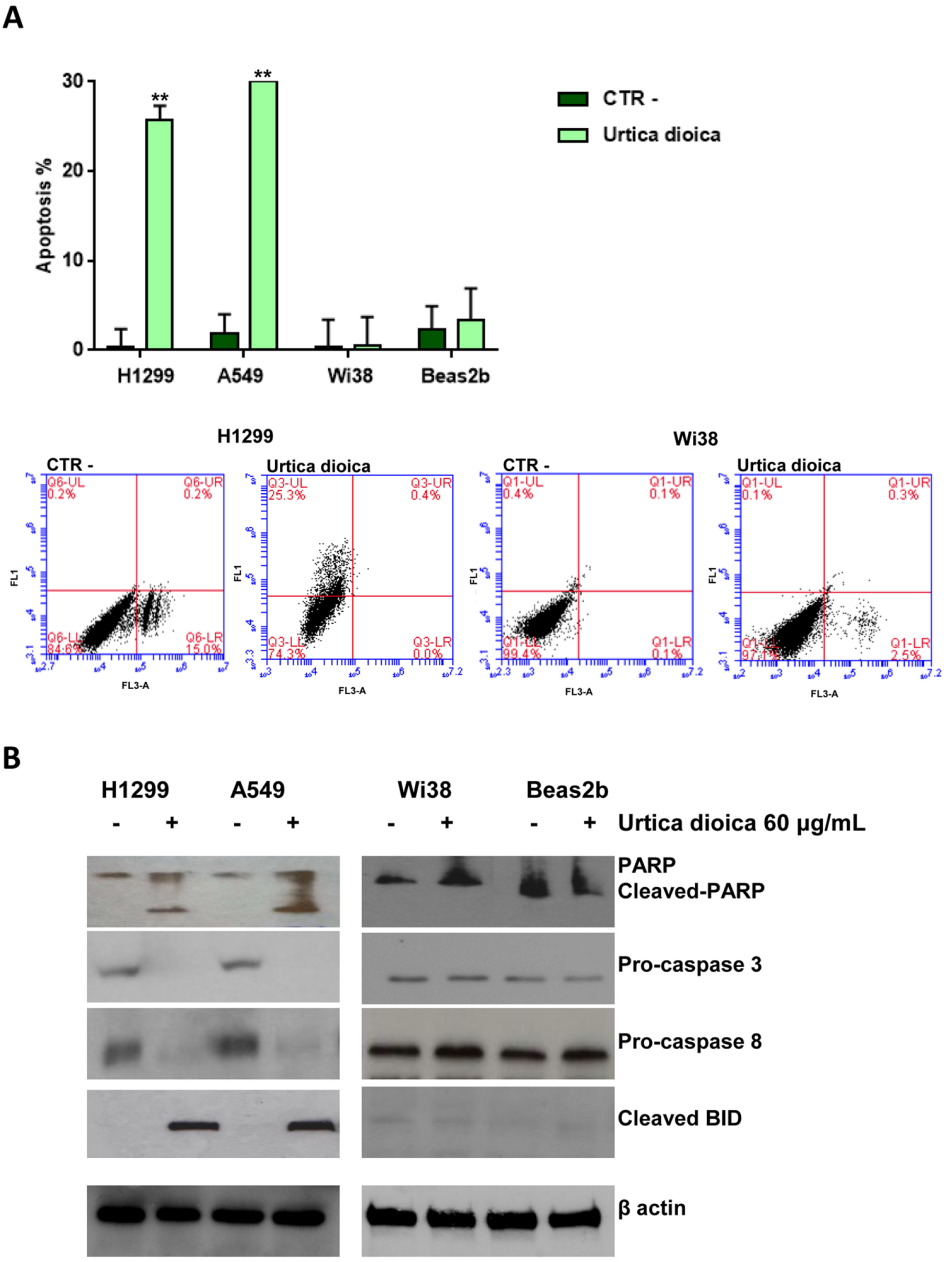
Induction of apoptosis in NSCLC (H1299 and A549) cell lines, normal bronchial epithelial (Beas2b) cells and human fibroblasts (Wi38). (**A**) Apoptosis was evaluated as described in Materials and Methods with Annexin V staining in H1299, A549, Beas2B and Wi38 cells after treatment with (60 μg/mL for 72 h) and without (CTR-) *Urtica dioica* extract. Representative dot plot diagrams of flow cytometric analysis of H1299 and Wi38 cell apoptosis are shown. Dot plot diagrams show the different stages of apoptosis. % indicated in the UL (Upper Left) quadrant represent cells positive for Annexin V and negative for 7AAD, considered as early apoptotic cells; % in UR (Upper Right) quadrant indicate cells positive for both Annexin V and 7AAD, showing the late apoptotic or necrotic cells population; % in LL (Lower Left) quadrant are negative for both markers and represent viable cells. Histogram of data expressed as percentage of both early and late apoptotic cells. Bars represent mean values obtained from three separate experiments. *P* values < 0.05 were considered as statistically significant (**) at Student-t test. (**B**) The Western Blot analyses, which were carried out using antibodies against PARP, (89)-cleaved-PARP fragment, pro-caspase3, pro-caspase 8 and cleaved BID, were performed on protein lysates from cell after the indicated treatment with (+) and without (−) *Urtica dioica* extract.

Subsequently, the induction of apoptosis in H1299 and A549 tumour cell lines in comparison with the non-tumour cell lines Wi38 and Beas2b was analysed, after the treatment with (60 μg/mL for 72 h) and without (CTR-) UD. As a result, approximately 25.3% for H1299 and 30% for A549 experienced apoptosis, meanwhile Beas2b and Wi38 did not show any post-treatment effects. Histogram data are expressed as a percentage of both early and late apoptotic cells, moreover representative dot plots diagrams of flow cytometric analysis of H1299 and Wi38 cell apoptosis are shown in the Fig. 2A.

Following this, the expression of the main apoptosis-related enzymes^20^ by using western blotting analysis was evaluated (see Fig. 2B and S1 in the Supplementary Information File). Decreased levels of the pro-caspase-3 and -8 indicated the activation of the proteolytic enzymes caspase-3 and -8, respectively. Furthermore, a concomitant increase of cleaved poly (ADP) ribose polymerase (c-PARP) (89 KDa) and truncated Bid (tBid) (15 KDa), that represented the correspondent substrates of caspase-3 and -8, undoubtedly confirmed this data.

**Figure 3:**
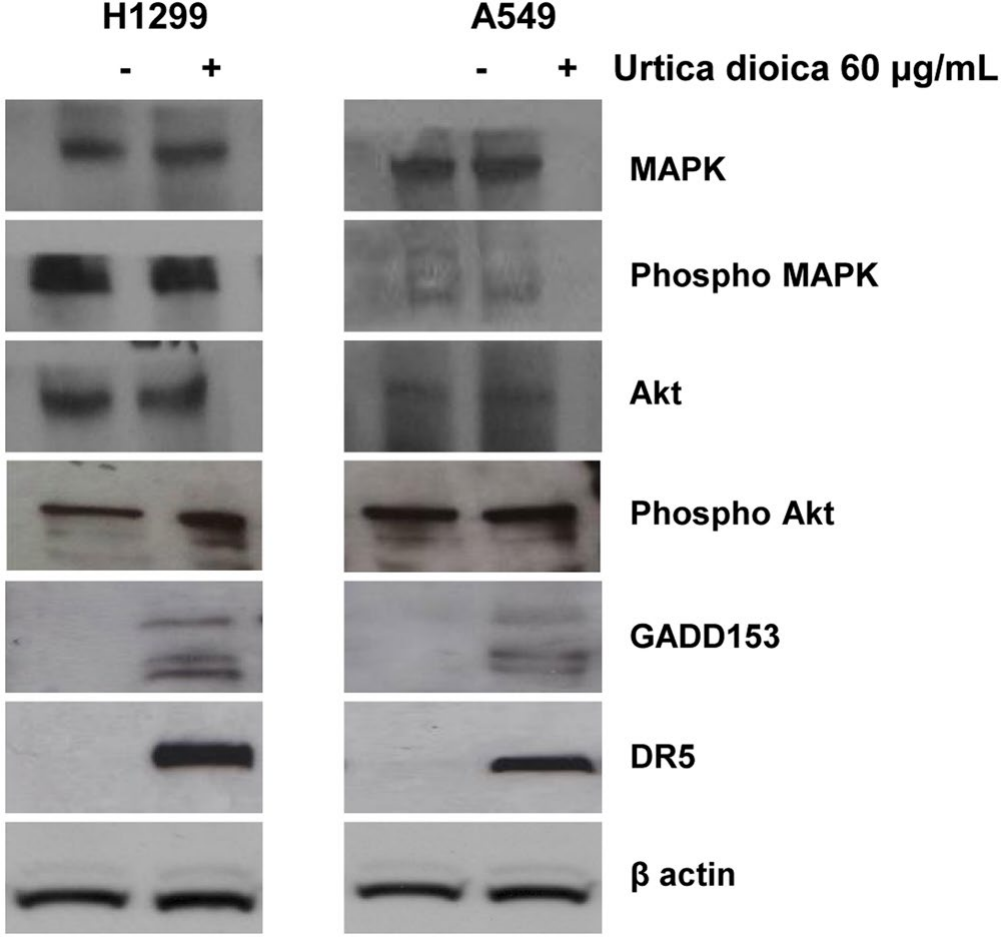
The expression levels of ER-stress related proteins were investigated on H1299 and A549 cancer cells following treatment with (+) and without (−) *Urtica dioica* extract (60 μg/mL) for 72 h. The Western Blot analyses were carried out using antibodies against MAPK, Phosho MAPK, AKT, Phosho AKT, GADD 153, DR5. β-actin was used as the loading control.

To identify the cellular signalling responsible for caspase activation, the MAPK and PI3K/Akt pathways were initially investigated, as they both play a pivotal role in regulating cell proliferation, apoptosis^20,21^ and cisplatin cytotoxicity^3^. The dysregulation of MAPK and PI3K/Akt pathways were found to impair cisplatin sensitiveness^22,23^ and this led to suppose that UD treatment could restore the activation of MAPK and PI3K/Akt pathways finally fostering apoptosis. In order to validate this hypothesis, the levels of phosphorylated MAPK (p44/42 MAPK) and Akt were analysed, since the activities of these proteins are modulated by phosphorylation. Nevertheless, the unphosphorylated/phosphorylated levels of MAPK and Akt had proven similar both before and after the treatment, suggesting that UD did not affect these proteins (See Fig. 3 and S2 in the Supplementary Information File).

Thus, other molecular and cellular determinants known to be responsible for caspase activation were studied. Previous works sustained that caspase-8 activity is likely to be externally stimulated by surface death receptors^24^, and therefore, these proteins were analysed, indicating the expression of the death receptor DR5 remarkably increased after UD treatment in both H1299 and A549 cells (Fig. 3). These findings revealed that UD promoted the extrinsic apoptotic pathway through DR5, which upon activation, triggered the caspase cascade along with the cleavage of cytosolic BID in tBID^20^. Moreover, DR5 up-regulation was known to be directly linked to the activation of the growth arrest and DNA damage-inducible gene 153 (GADD153), also known as the C/EBP homologous transcription factor (CHOP), which was considered a marker of endoplasmic reticulum (ER) stress^25,26^. Therefore, GADD153 levels were examined, displaying that this protein was evidently up-regulated in H1299 and A549 cells after 72 h of incubation with UD (Fig. 3).

Taken together, the findings supported a mechanistic scenario in which UD treatment induced ER-stress by up-regulating GADD153. In turn, this event resulted in the overexpression of DR5, which directly promoted the extrinsic apoptotic pathway and indirectly stimulated the mitochondria apoptotic machinery via BID activation.

### The synergistic anti-proliferative effect of *Urtica dioica* extract and cisplatin against human lung cancer cells

**Figure 4:**
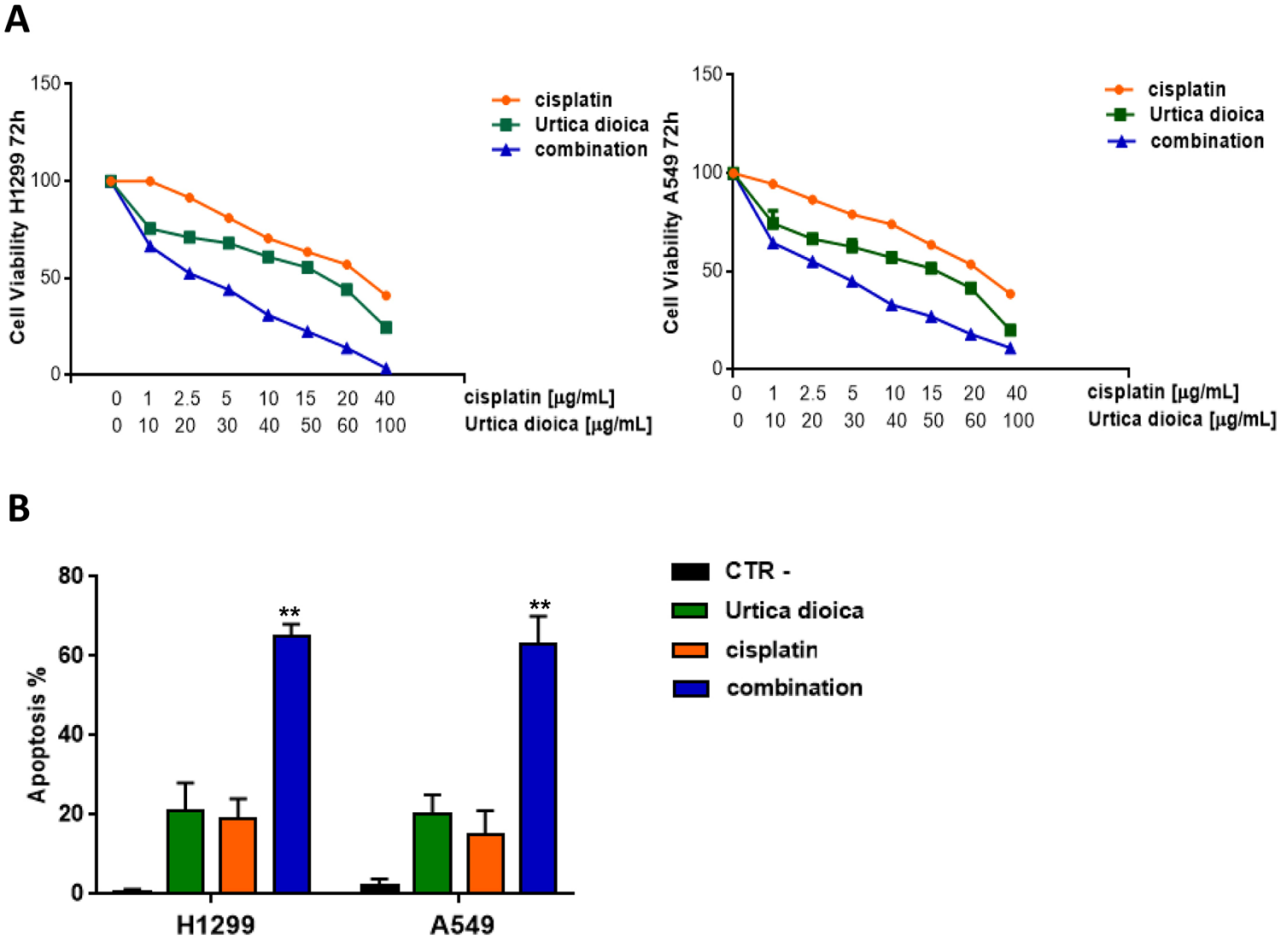
Effects of the combination of *Urtica dioica* extract and cisplatin on NSCLC (H1299 and A549) cell lines. (**A**) Cell proliferation analysis performed by MTT assay in H1299 and A549 after treatment with the indicated doses of *Urtica dioica* and cisplatin. The results are the average ± sd of three independent experiments, each done in triplicate. All the used doses are statistically significant as determined by the Student-t test (**P ≤ 0.01). For sake of simplicity asterisks (**) are not reported in the graph. (**B**) Apoptosis evaluated in H1299 and A549 after treatment with the indicated doses of nettle and cisplatin. Histogram of data expressed as percentage of both early and late apoptotic cells. Bars represent mean values obtained from three separate experiments. Statistically significant data are evidenced with asterisks (**P ≤ 0.01).

To extend the pre-clinical observations, it was evaluated whether UD was able to improve the sensitivity of H1299 and A549 cell lines to cisplatin. For this purpose, cells were treated with cisplatin and/or UD (Fig. 4A). As expected, treatment with cisplatin alone, weakly inhibited cell proliferation (IC_50_ = 32.012 ± 0.004 and 22.156 ± 0.003 µg/mL, for A549 and H1299, respectively), whereas, the co-treatment of cisplatin and UD exhibited a notable anti-proliferative synergistic effect in both H1299 and A549 cells (Fig. 4A). The administration of cisplatin at 2.5 μg/mL or UD at 20 μg/mL caused a comparable effect that was approximatively accountable for 20% apoptotic rate; conversely, the combination of these dramatically raised the apoptotic percentage reaching approximately 65% (Fig. 4B). These results indicated that UD extract synergised with cisplatin, radically improving the sensitivity of H1299 and A549 cells to cisplatin treatment.

### 1D and 2D-NMR spectroscopy investigation of *Urtica dioica* extract

The encouraging biological results resulted in the performance of a comprehensive NMR analysis with the aim of unveiling the main secondary metabolites present in the plant extract.

Metabolites were identified by comparing peak chemical shifts to those found in literature and in Human Metabolome Database (HMDB). Furthermore, as spectral overlaps of the ^1^H resonances in 1D spectra often seriously limited the unambiguous identification of certain metabolites, we also carried out an extensive 2D NMR analysis of the plant extract.

**Figure 5:**
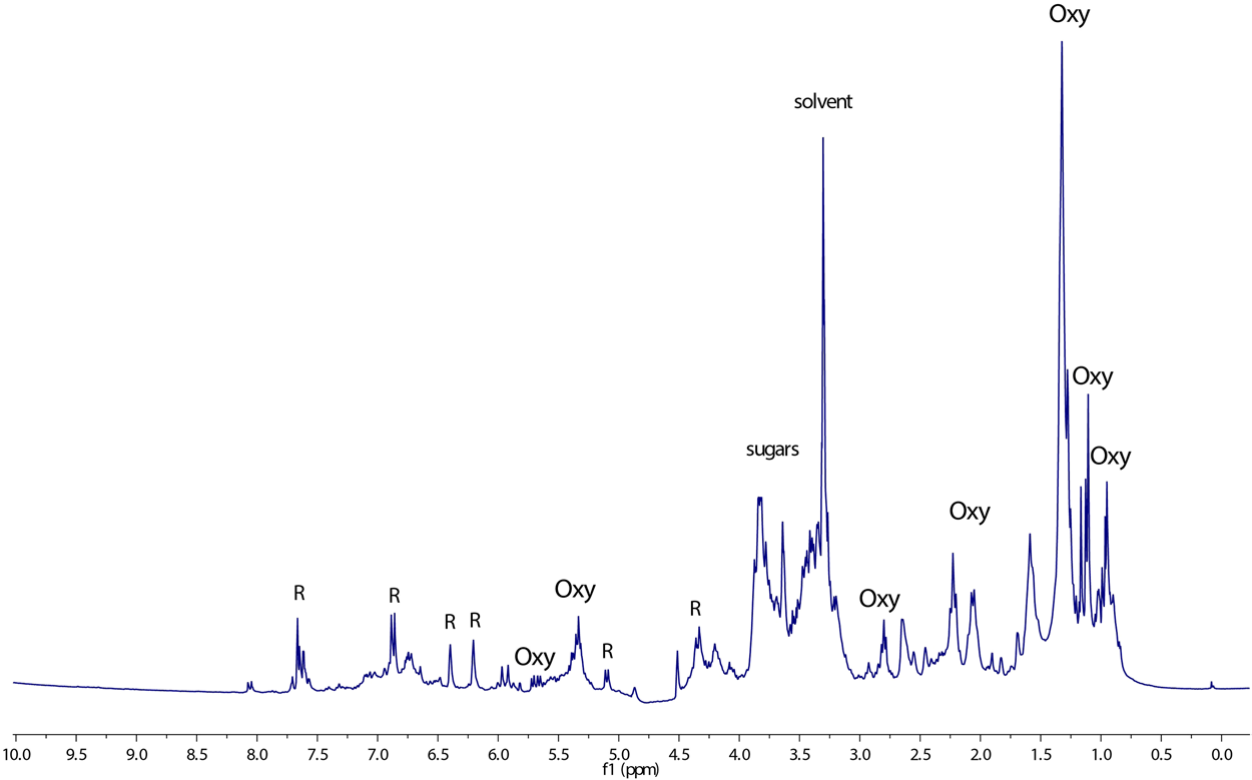
^1^H NMR spectrum of *Urtica dioica* crude extract recorded in methanol*-d*_4_/phosphate buffer (1:1). R = rutin, Oxy = oxylipins, sugar = glycosidic moiety of rutin.

The ^1^H NMR spectrum of UD (Fig. 5) displayed peculiar chemical shift values of flavonoids evident in the aromatic region of the spectrum; in particular, two meta-coupled doublets at δ_H_ 6.39 (δ_C_ 93.5) and δ_H_ 6.20 (δ_C_ 98.7) were clearly detectable. These signals, thanks to the CIGAR-HMBC correlations (see Figure S3 in the Supplementary Information File) along with available literature data^27^, were assigned to quercetin. Moreover, two signals at δ_H_ 5.01 and 4.53 also correlated in CIGAR-HMBC experiment with the C-3 carbon (δ_C_ 135.2) and the methylene carbon of glucose (δ_C_ 68.1), respectively. This data proved to be in line with the presence of quercetin-3-O-rutinoside, a flavonol glycoside known as rutin, which has already been reported as a representative constituent of *U. dioica* flowers^28^.

**Figure 6:**
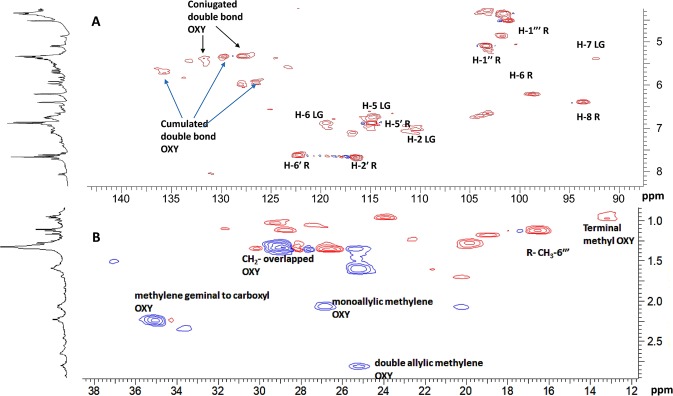
(**A**) Expanded olefinic and (**B**) aliphatic region of HSQC experiment of *Urtica dioica* extract. R = rutin. LG = lignan. OXY = oxylipins

**Figure 7:**
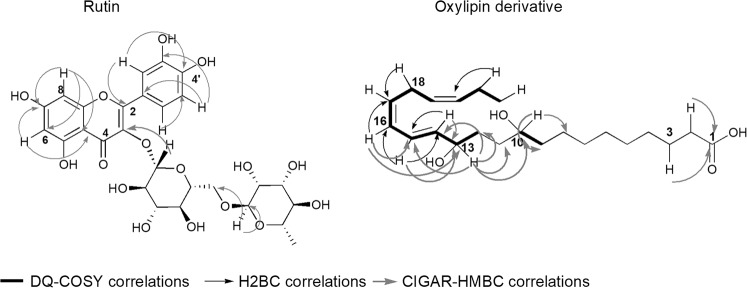
Selected CIGAR-HMBC, DQF-COSY and H2BC correlations of rutin and oxylipin derivatives detected in *Urtica dioica* crude extract.

In the region of the ^1^H-NMR spectrum included between 6.00 and 5.00 ppm, several overlapped protons were evident. Furthermore, two methylene triplets (Figs 5 and 6) at δ_H_ 2.80 (δ_C_ 25.2) and 2.23 (δ_C_ 35.0) along with overlapped methylene protons at 2.06 (δ_C_ 26.8 and 20.3), 1.59 (δ_C_ 25.1), 1.32 (δ_C_ 29.0) and the methyl triplet at 0.95 (δ_C_ 13.1) supported the presence of omega-3 fatty acids. These data were in agreement with previous works, which identified α-linoleic acid and its derivatives as the pre-dominant fatty acids of *U.dioica* leaf extract^29^. The combination of 2D-NMR techniques, especially DQF-COSY, H2BC and HSQCTOCSY (see Figure S4 in the Supplementary Information File), allowed the assignment of almost all overlapped H-atom and C-atom signals. Specifically, DQF-COSY homocorrelations (see Figure S5 in the Supplementary Information File) led to the identification of two types of spin system: CH_3_-CH_2_-CH=CH-CH_2_-CH=CH and CH=CH=CH-CH(O) as shown in Fig. 7. The former spin system confirmed the presence of an omega-3 fatty acid, while the latter suggested that this compound included a site of hydroxylation. This was further supported by several works that previously demonstrated the presence of oxylipins (polyunsaturated oxidised fatty acid) in *U. dioica*^30,31^. Moreover, in the CIGAR-HMBC experiment, the methine proton at δ_H_ 5.70 was heterocorrelated with carbon at δ_C_ 128.5 that bonded to the proton at δ_H_ 5.94. This latter signal was heterocorrelated with the olefinic carbon at δ_C_ 136.7 as well as with an oxygenated C-atom at δ_C_ 77.0 (see Figure S3 in the Supplementary Information File). In turn, this was heterocorrelated with the protons at δ_H_ 2.68, 2.26, 1.36. Specifically, the carboxyl carbon at δ_C_ 180.1 displayed cross peaks with the methylene protons at δ_H_ 2.32 (δ_C_ 34.1), which, in turn, heterocorrelated with carbons at δ_C_ 29.1 (δ_H_ 1.29) and 24.6 (δ_H_ 1.64) (see Figure S3 in the Supplementary Information File). Altogether, the data suggested the presence of hydroxyl polyunsaturated fatty acids. However, due to the lipid nature of these metabolites, it was not possible to define the exact structures of the above-mentioned oxylipins in the crude extract.

Besides flavonols and oxylipins, less intense signals, overlapped in the^1^H-NMR spectrum, were detectable in the 2D NMR experiments and ascribable to less abundant UD metabolites. Most of these signals were attributed to lignan-type molecules. Specifically, thanks to the HSQC experiment (Fig. 6), six aromatic hydrogen signals, which belonged to two 1,2,4-trisubstituted phenyl groups were detected. Moreover, key CIGAR-HMBC correlations indicated the presence of two guaiacyl groups linked to tetrahydrofuran moiety through an oxymethine (δ_H_ 4.73/δ_C_87.1) and an oxymethylene^32^. This data supported the presence of olivil derivatives, tetrahydrofuranic lignans that had already been identified as a constituent of *Urtica triangularis*^33^.

### Identification and isolation of the major components of *Urtica dioica* that contribute to the cytotoxicity of the active plant extract

Subsequently, it was investigated whether the major components of the UD contributed to its selective cytotoxic effect on NSCL cells. For this purpose, a targeted fractionation of UD was performed using different chromatographic techniques. Initially, the plant extract was portioned between ethyl acetate (UD1) and water (UD2): rutin was isolated from both UD1 and UD2, while an oxylipins’ enriched fraction was obtained exclusively from UD1. The pure compound rutin was analysed trough 1D and 2D NMR (see Fig. 7 and S6 in the Supplementary Information File); thus, its structure was confirmed by comparing NMR data with those available in literature^34^ and with the in-house NMR library. Likewise, the oxylipins’ enriched fraction was firstly analysed trough 1D and 2D NMR. In the ^1^H-spectrum, overlapped signals resonating in the range 0.88–1.00 ppm and 4.40–3.80 ppm along with two triplets at δ_H_ 5.96 and 5.95 and two double doublets at δ_H_ 5.64 and 5.63. These signals were in agreement with those detected in the plant crude extract and supported the presence of omega 3-oxylipins, whose basic skeleton was further confirmed by 2D NMR correlations (see Figures S7 and S8 in the Supplementary Information File).

**Figure 8:**
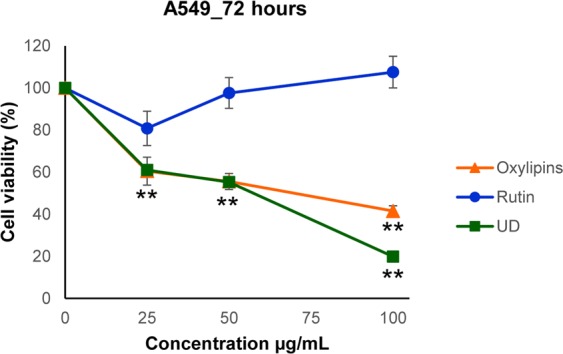
Cytotoxic effect of rutin, oxylipins and UD extract on A549 cell line. Cell proliferation was evaluated 72 h after treatment by MTT assay; results are reported as mean ± sd of 3 independent experiments, each done in triplicate. Inhibition of cell growth exerted by Oxylipins and UD extract vs control was statistically significant at Student- t test (**P ≤ 0.01).

Subsequently, the cytotoxicity of these compounds on A549 cell line were assessed through the MTT assay testing four different doses (25, 50, 75 and 100 µg/mL) at 72 hours. These experiments demonstrated that rutin did not show any cytotoxic effect, while the oxylipins’ enriched fraction recapitulated the effect of the crude plant extract (Fig. 8).

Due to the interesting biological results, the main aim was to elucidate the structure of the main oxylipin present in the above-mentioned mixture. However, the severe overlaps in the NMR spectra made again not feasible the unambiguous characterisation of these metabolites. Therefore, an HPLC analysis was carried out by identifying and isolating the principal oxylipin of the fraction. Its structure was then elucidated thanks to a combination of ESI Q-TOF HRMS and NMR techniques. The ESI Q-TOF HRMS spectrum in the negative mode showed a pseudomolecular ion [M-H]^−^ at m/z 365.2706, giving the molecular formula C_22_H_38_O_4_ for this oxylipin. Meanwhile, the positive mode of the ESI Q-TOF HRMS spectrum displayed a sodium adduct at m/z at 389.2649 along with two fragments at m/z 349.2723 and 331.2623. The two last peaks suggested the consecutive loss of two molecules of H_2_O (see Figure S9 in the Supplementary Information File). These data were in good agreement with the HSQC spectrum (see Figure S10 and Table S1 in the Supplementary Information File), in which the presence of two carbinolic methins at δ_C_ 69.2 (δ_H_ 4.01) and δ_C_ 72.9 (δ_H_ 4.09) was evident. This last proton was heterocorrelated with the C10 (δ_C_ 69.2), two methylene carbons C11 (δ_C_ 28.3) and C12 (δ_C_ 36.1), and two methin carbons C14 (δ_C_ 137.8) and C15 (δ_C_ 126.1). This latter in the H2BC experiment showed a ^2^J correlation with the triplet at δ_H_ 5.99, which correlated with the olephinic carbon C17 at δ_C_ 131.5 (Fig. 7). In turn, this carbon displayed a cross peak with the double allylic protons H18 at δ_H_ 2.95 (δ_C_ 26.6). Moreover, the HMBC experiment evidenced key correlations among a series of methylene protons (resonating at δ_H_ 2.24, 2.05, 1.6 and 1.34) and the carbonyl carbon at δ_C_ 182.2. This data was further confirmed by the tandem MS analysis of the pseudomolecular ion [M - H]^−^, which showed two fragment ions at m/z 347.1905 and 303.2093, indicating the loss of H_2_O and H_2_O along with CO_2_, respectively. Altogether, these data were in agreement with the presence of 10,13-dihydroxydocosa-12,16,19-trienoic acid.

## Discussion

For thousands of years, nature was considered an ideal source of pharmacological agents; as a result, the majority of antibiotics and anti-cancer medicines were composed or at least derived from natural products^35^. In particular, medicinal plants provided a plethora of innovative chemotherapeutic agents that revolutionised the anti-cancer therapies clearly improving patient treatment outcome^6^.

Recently, an increasing number of studies documented the anti-inflammatory and the anti-tumour properties of *U.dioica* L. To date, it has been understood that UD exerted beneficial effects in the treatment of benign prostatic hyperplasia (BPH)^16^ and cytotoxic activity against human prostate cancer cells^15,34^. Evidence reported that these properties might be related to the capability of the plant extract to inhibit the α5-reductase enzymes^16,36^. Additional investigations showed that this species fostered apoptosis in breast cancer models^17^, and the combination of paclitaxel and UD significantly enhanced the effect of the paclitaxel alone against the MDA-MB-468 human breast cancer cell line^37^. However, no previous reports investigated the antineoplastic effect of UD in NSCLC.

Here, the methanol extract of *U.dioica* (UD) proved to reduce tumor cell proliferation in NSCLC cells and identify the major chemical components of the plant extract. For this investigation H460, H1299, A549 and H322 cells were selected as an ideal model to study EGFR wild typ e NSCLs with a low cisplatin sensitivity^4^.

Our findings highlighted that UD differentially inhibited cell proliferation in A549, H1299, H460 and H322 cells (IC_50_ values: 52.3 and 47.4 µg/mL, for H1299 and A549 respectively; IC_50_ values: 84.3 and 78.3 µg/mL for H460 and H322 respectively), while it did not show substantial toxicity in normal bronchial epithelial cells (Beas2B) and lung fibroblasts cells (Wi38). In particular, a more evident response to treatment in terms of reduction of cell viability, is mainly found in cells A549 and H1299 while the other two cell lines undergo a lower reduction of viability. For this reason, attention was focused only on the most sensitive UD cell lines, comparing them with healthy lines.

Indeed, in Beas2B and Wi38 cells the 50% inhibition of the cell viability was not reached even at the highest dose of treatment employed (UD = 100 µg/mL). As NCSLC patients that do not harbor genetic alterations in EGFR, are mainly treated with cisplatin-based therapies^5^, the co-treatment of UD and cisplatin was performed in order to understand whether the plant extract improved the effect of the treatment with cisplatin alone. As a result, it was demonstrated that the aforementioned co-treatment had a notable positive synergistic effect, remarkably sensitising H1299 and A549 to cisplatin.

Furthermore, this study provided a mechanistic framework that at a molecular level highlighted the mechanisms underlying the selective anti-proliferative effect of the plant extract. It was revealed that UD treatment promoted endoplasmic reticulum (ER) stress via GADD153 and DR5 activation, finally inducing apoptosis. ER is the organelle in which the synthesis and folding of secretory properties were carried out. However, under certain physiological and pathological conditions, protein folding could be dramatically impaired leading to ER stress^38^. As a consequence, specific ER stress response pathways were activated; in particular, apoptosis was enabled when a high load of misfolded protein occurred^39^. GADD153 encoded a member of the CCAAT/enhancer-binding protein family and acted as an inhibitor or activator of transcription, leading to apoptosis^21^. According to our findings, its expression was found increased in ER stress induced-apoptosis and it was linked to the activation of DR5, a cell surface receptor that stimulated the extrinsic apoptotic pathway^25^. It is well known that the DR5-derived signal was transduced inside the cell through the initiator caspase 8, which, in turn, activated the caspase cascade and BID^20^. In line with this, we found that the DR5 overexpression is accompanied by an increase of the caspase effectors and truncated BID (tBid). tBID is a pro-apoptotic fragment that accumulated at the outer mitochondrial membrane and activated the apoptotic mitochondrial apparatus^26^, linking the extrinsic with the intrinsic apoptotic pathways, thereby amplifying the signal of programmed cell death.

Previous phytochemical investigations of UD described this species as a source of valuable chemical compounds, including phenols, carotenes, essential oils and fatty acids^14^. In this study, thanks to an extensive NMR analysis, rutin and oxylipins were identified as the main secondary metabolites present in the plant mixture. This analysis guided a targeted UD fractionation that allowed the rapid isolation of a rutin and an oxylipins’ enriched fraction circumventing time-consuming procedures^40^. In contrast with other previous works^41^, that showed the anti-proliferative activity of rutin against diverse human cancer models including NSCL cells, it was demonstrated that this metabolite did not exert any effect. Meanwhile, it was proved that oxylipins strictly resembled the activity of the crude extract, indicating that these contributed to the anti-proliferative activity of UD. Oxylipins are a large family of metabolites derived from polyunsaturated fatty acids and produced by animals, microorganism, fungi, algae and plants. Their structure variability mirrors the wide range of functions that they carried out in diverse organisms and biological contexts. Naturally-derived oxylipins are often endowed with similar or identical structures compared with the oxylipins found in human tissues^42^, and thus, these molecules are considered biochemicals of high value for the search of new pharmacological agents^43,44^. Supporting this data, previous works highlighted the cytotoxic potential of several oxylipins derived from diverse natural sources^45,46^, and due to these antineoplastic properties, Romano *et al*.^47^ synthesised pro-apoptotic compounds inspired by diatom oxylipins. Interestingly, several oxylipins have been already identified from *U. dioica*. Interestingly, a commercially available lipophilic leaf extract of *U. dioica* (Hox-alpha) has been developed and it has been known to include a high concentration of the oxylipin 13-S-hydroxy-9Z, 11E, 15Z-octadecantrienoic acid. Hox-alpha was extensively studied for its potential use as adjuvant agent in the treatment of chronic inflammatory disease such as rheumatoid arthritis^31^.

In conclusion, here, it was discovered that UD selectively killed NSCLC cells, by promoting ER-mediated apoptosis. An extensive NMR analysis revealed the major components of the active plant extract and guided a targeted phytochemical study that lead to obtain rutin along with an oxylipins’ enriched fraction. The cytotoxic assessment of these compounds revealed that oxylipins contributed to the activity of UD. These insights pave the way to future experiments that will focus on the oxylipins’ enriched fraction with the ultimate goal of identifying new lead compound candidates to develop new agents against NSCLC with low cisplatin sensitivity.

## Materials and Methods

### Plant material

*Urtica dioica* L. leaves were collected in a garrigue on the calcareous hills of Durazzano, (41°3′N, 14°27′E; southern Italy) in the vegetative state and identified by Dr. Assunta Esposito of the Dept. of Environmental, Biological and Pharmaceutical Sciences and Technologies of the “Luigi Vanvitelli” University of Campania. A voucher specimen was deposited at the Department Herbarium. *Urtica dioica* leaves were harvested and immediately frozen in N_2_ liquid in order to avoid unwanted enzymatic reactions and stored at −80 °C up to the freeze-drying process. Once freeze dried, they were powdered in liquid nitrogen and stored at −20 °C until the extraction process was carried out.

### Extraction procedure for metabolomic analysis

An aliquot (50 mg) of freeze-dried and powdered plant material was transferred to a 2 mL microtube. 1.5 mL of phosphate buffer (Fluka Chemika, Buchs, Switzerland; 90 mM; pH 6.0) in D_2_O (Cambridge Isotope Laboratories, Andover, MA,USA)-containing 0.1% w/w trimethylsilylpropionic-2,2,3,3-d4 acid sodium salt (TMSP, Sigma–Aldrich, St. Louis, MO, USA)- and CD3OD (Sigma–Aldrich, St. Louis, MO, USA) (1:1) were added to the samples. The mixture was vortexed at room temperature for 1 min, ultrasonicated (Elma Transsonic Digital, Hohentwiel, Germanys) for 40 min, and centrifuged (Beckman Allegra™ 64 R, F2402H rotor; Beckman Coulter, Fullerton,CA, USA) at 13,000 rpm for 10 min. Finally, a volume of 0.65 mL was transferred to a 5-mm NMR tube and analysed by NMR^48^.

### Nuclear magnetic resonance spectroscopy (NMR) experiments

NMR spectra were recorded at 25 °C on a Varian Mercury Plus 300 Fourier transform NMR spectrometer operating at the frequencies 300.03 MHz for ^1^H and 75.45 MHz for ^13^C. CD_3_OD was used as the internal lock.

Each ^1^H NMR spectrum consisted of 256 scans with the following parameters: 0.16 Hz/point, acquisition time (AQ) = 1.0 s, relaxation delay (RD) = 1.5 s, 90° pulse width (PW) = 13.8 μs. A pre-saturation sequence was used to suppress the residual H_2_O signal. FIDs were Fourier transformed with LB = 0.3 Hz. The resulting spectra were manually phased and baseline-corrected and calibrated to TMSP at 0.0 ppm using ^1^H NMR processor (ACDLABS 12.0).

^1^H-^1^H correlated spectroscopy (COSY), double quantum filtered COSY (DQF-COSY) spectra heteronuclear single quantum coherence (HSQC) and heteronuclear multiple bond correlation (HMBC) spectra were recorded. COSY and DQF-COSY spectra were acquired with a 1.0 s relaxation delay and 2514 Hz spectral width in both dimensions. The window function for COSY and DQF-COSY spectra was sine-bell (SSB = 0). Heteronuclear 2 bond correlation (H2BC) spectra were obtained with T = 30.0 ms, and a relaxation delay of 1.0 s; the third-order low-pass filter was set for 130 < ^1^J(C,H) < 165 Hz. HSQC and HMBC spectra were obtained with a 1.0 s relaxation delay and 3140 Hz spectral width in f2 and 18116 Hz in f1. Qsine (SSB = 2.0) was used for the window function of the HMBC. The optimised coupling constants were 140 Hz for HSQC and 8 Hz for HMBC. Constant time inverse-detection gradient accordion rescaled heteronuclear multiple bond correlation spectroscopy (CIGAR–HMBC) spectra (8 > ^n^*J*_(H,C)_ > 5) were acquired with the same spectral width used for HMBC. Heteronuclear single-quantum coherence - total correlation spectroscopy (HSQC-TOCSY) experiments were optimised for ^n^*J*_(H,C)_ = 8 Hz, with a mixing time of 90 ms.

### Extraction procedure for biological assay

1.67 g of lyophilised leaves and 50 mL of a mixture H_2_O/MeOH (1:1) were mixed together, extracted by ultrasound assisted extraction for 40 minutes and then centrifuged at 13000 rpm for ten minutes. The surnatant was completely removed and the solvent distilled by rotavapor, giving a crude extract, that was subsequently purified even further with a SEP-PAK C18 cartridge in order for the plant extract to become enriched in secondary metabolites.

### The isolation of active metabolites

Dried *urtica dioica* leaves (44.0 g) were powdered and subjected to five cycles of ultrasound assisted extraction with a MeOH/H_2_O (1:1) solution, obtaining a crude extract (47.6 g). This extract, once dissolved in H_2_O, was fractionated by liquid–liquid extraction using EtOAc as an extracting solvent. The water fraction was chromatographed by Sephadex H20 CC, eluted with a decreasing degree of polarity H_2_O:MeOH, finally obtaining 11 fractions. Among these, fraction 10 was in turn purified by TLC with 14:6:1 (CHCl_3_:MeOH:H_2_O) as eluting solution, giving Rutin (10 mg). The organic fraction (1.6 g) was separated by liquid–liquid extraction with a MeOH/hexane (1:2) solution, giving a polar and an apolar fractions, respectively. This latter, chromatographed by RP-18 CC eluting with a solution with a decreasing degree of polarity H_2_O: MeOH produced five fractions. Fraction 1 was identified as rutin (25 mg) while fraction 2, that consisted of oxylipins (18 mg), was chromatographed through LUNA-RP 18 HPLC [H_2_O/MeOH/MeCN (2:5:3)] providing the main oxylipin (11.0 mg).

### Cell lines and chemicals

The human NSCLC H460, H1299, A549 and H322 were provided by the American Type Culture Collection (Manassas, VA) and were maintained in RPMI 1640 supplemented with 10% fetal bovine serum (FBS; Life Technologies, Gaithersburg, MD) in a humidified atmosphere with 5% CO_2_. Normal bronchial epithelial cells, Beas2B cells, and lung fibroblast, Wi38, were kindly provided by Dr Clementina Sansone, Stazione Zoologica Anton Dohrn, Naples, Italy.

Cisplatin was purchased from Selleck Chemicals (Selleckchem). Annexin V-FITC apoptosis detection kit was obtained from BD Biosciences Pharmingen, CA, USA. All other chemicals were purchased from Sigma–Aldrich, MO, USA.

### Cell proliferation assay

The human NSCLC H460, H1299, A549, H322, the normal bronchial epithelial cells, and lung fibroblast, Beas2B and Wi38, respectively, were seeded in 96-multiwell plates (1 × 10^3^ cells/well) and treated with different doses of *Urtica dioica* extract. Cell proliferation was measured with the 3-(4,5-dimethylthiazol-2-yl)-2,5-diphenyltetrazolium bromide (MTT) assay. The drug concentrations required to inhibit cell growth by 50% (IC_50_) were determined by interpolation from the dose-response curves. The results are the average ± sd of three independent experiments, each done in triplicate. Synergism was calculated with ComboSyn software, ComboSyn Inc., Paramus, NK. 07652 USA.

### Western blotting analysis

Cancer cells were lysed with Tween-20 lysis buffer (50 mmol/L HEPES, pH 7.4, 150 mmol/L NaCl, 0.1% Tween-20, 10% glycerol, 2.5 mmol/L EGTA, 1 mmol/L EDTA, 1 mmol/L DTT, 1 mmol/L phenyl- methylsulfonylfluoride, and 10 μg/mL of leupeptin and aprotinin) and sonicated. Equal amounts of protein were analysed by SDS-PAGE. Subsequently, proteins were transferred to nitrocellulose membranes and analysed by specific primary antibodies diluted 1:1000 in blocking solution. Primary antibodies used for western blot analysis were against Parp, caspase-8, caspase-3, BID, MAPK42/44, phospho-MAPK42/44, AKT, phospho-AKT, GADD153, DR5 and β-actin were obtained from Cell Signalling Technology; The following secondary antibodies from Bio-Rad were used: goat anti-rabbit IgG, rabbit anti-mouse IgG and monoclonal anti β-actin antibody from Sigma Chemical Co. Proteins were detected via incubation with horseradish peroxidase – conjugated secondary antibodies and ECL chemiluminescence detection system.

### Cell Cycle Distribution and Apoptosis Assessment

In order to assess the effects on cell cycle and the induction of apoptosis, NSCLC cell lines were plated in 100-mm tissue culture dishes (Becton Dickinson) and treated with the indicated concentrations of either *Urtica dioica*, cisplatin alone, or in combinations as described above. After another 24 hours, both adherent and detached cells were harvested. Flow cytometric analysis of apoptotic cell death was done on cell pellets that were fixed in 70% ethanol, washed in PBS, and mixed with RNase (Sigma) and propidium iodure (Sigma) solution as reported previously^37^. DNA content was analysed by a FACS can flow cytometer (Becton Dickinson) coupled with a Hewlett-Packard computer, and the percent of apoptotic cells was calculated by gating the hypodiploid region on the DNA content histogram using the LYSYS software (Becton Dickinson). Cell cycle data analysis was done using the CellFit software (Becton Dickinson). Apoptosis was detected by flow cytometry using an Annexin V/7AAD double staining (thermo fisher). Annexin V reveals the phosphatidylserine exposure of the altered plasma membrane; 7AAD is a vital dye, impermeant to live and early apoptotic cells, and was used to distinguish death cells. Briefly, cells were harvested, washed twice with PBS and then resuspended in 1X annexin-binding buffer at a concentration of 1 × 106 cells/mL according to the manufacturer’s instruction. Cells were stained adding Annexin V-FITC and 7AAD (100 μg/mL) into cell suspension and incubated in the dark for 15 min at room temperature. After the incubation, cells were analysed by flow cytometry within 30 min. All early apoptotic cells (Annexin V-positive, 7AAD–negative), necrotic/late apoptotic cells (double positive), as well as living cells (double negative) were detected by ACCURI C6 flow cytometer and subsequently analysed by ACCURI C6 software (Becton Dickinson). Argon laser excitation wavelength was 488 nm; Annexin V-FITC was detected by FL-1 channel, 7AAD by FL-3 channel.

### Statistical analysis

Results are expressed as means ± s.d. from three or more independent experiments. Differences between groups were assessed by one-way analysis of variance (ANOVA) followed by the Student t-test. For all analyses, *P* values represent 2-sided tests of statistical significance effects.

## Supplementary information

Supplementary information file
