# Supplementary material for: Urtica dioica L. inhibits proliferation and enhances cisplatin cytotoxicity in NSCLC cells via Endoplasmic Reticulum-stress mediated apoptosis
Source: Sci Rep. 2019 Mar 21;9:4986. doi: 10.1038/s41598-019-41372-1 (PMC6428841; doi:10.1038/s41598-019-41372-1)
Supplement: Supplementary file 1 — Supplementary information file [file 41598_2019_41372_MOESM1_ESM.pdf]

Urtica dioica L. inhibits proliferation and enhances cisplatin cytotoxicity in NSCLC cells *via* Endoplasmic Reticulum-stress mediated apoptosis

Brigida D'Abrosca*[1,] Vincenza Ciaramella[2§], Vittoria Graziani[1§], Federica Papaccio[2], Carminia Maria Della Corte[2], Nicoletta Potenza[1], Antonio Fiorentino[1], Fortunato Ciardiello[2] and Floriana Morgillo[2]

[1]Dipartimento di Scienze e Tecnologie Ambientali Biologiche e Farmaceutiche –DiSTABiF, Università degli Studi della Campania "Luigi Vanvitelli", via Vivaldi 43 I-81100, Caserta, Italy

[2]Dipartimento di Medicina di Precisione, Università degli Studi della Campania "Luigi Vanvitelli" 80131 Via Pansini, 5 - Napoli, Italy

[§]Vincenza Ciaramella and [§]Vittoria Graziani contributed equally to this work. Correspondence and requests for materials should be addressed to *Brigida D'Abrosca (brigida.dabrosca@unicampania.it)

Figure S1. Western Blot analyses of the proteins involved in apoptosis of NSCLC (H1299 and A549) cell lines, normal bronchial epithelial (Beas2b) cells and human fibroblasts (Wi38)

Figure S2. The expression levels of ER-stress related proteins

Figure S3. CIGAR experiment of *Urtica dioica* crude extract. R= rutin. OXY=oxylipins

Figure S4. HSQC-TOCSY experiment of *Urtica dioica* crude extract

Figure S5. DQCOSY experiment of *Urtica dioica* crude extract. R= rutin. OXY=oxylipins

Figure S6. HMBC experiment of rutin

Figure S7. ^{1}H-NMR spectrum of oxylipins' enriched fraction

Figure S8. H2BC experiment of oxylipins' enriched fraction

Figure S9. ESI Q-TOF MS spectrum of main oxylipin acquired in negative (A) and positive ion mode (B)

Figure S10. HSQC experiment of the main oxylipin

Table S1. NMR data of oxylipin recorded in CD_3OD. ^{1}H and ^{13}C are measured in ppm

Figure S1.

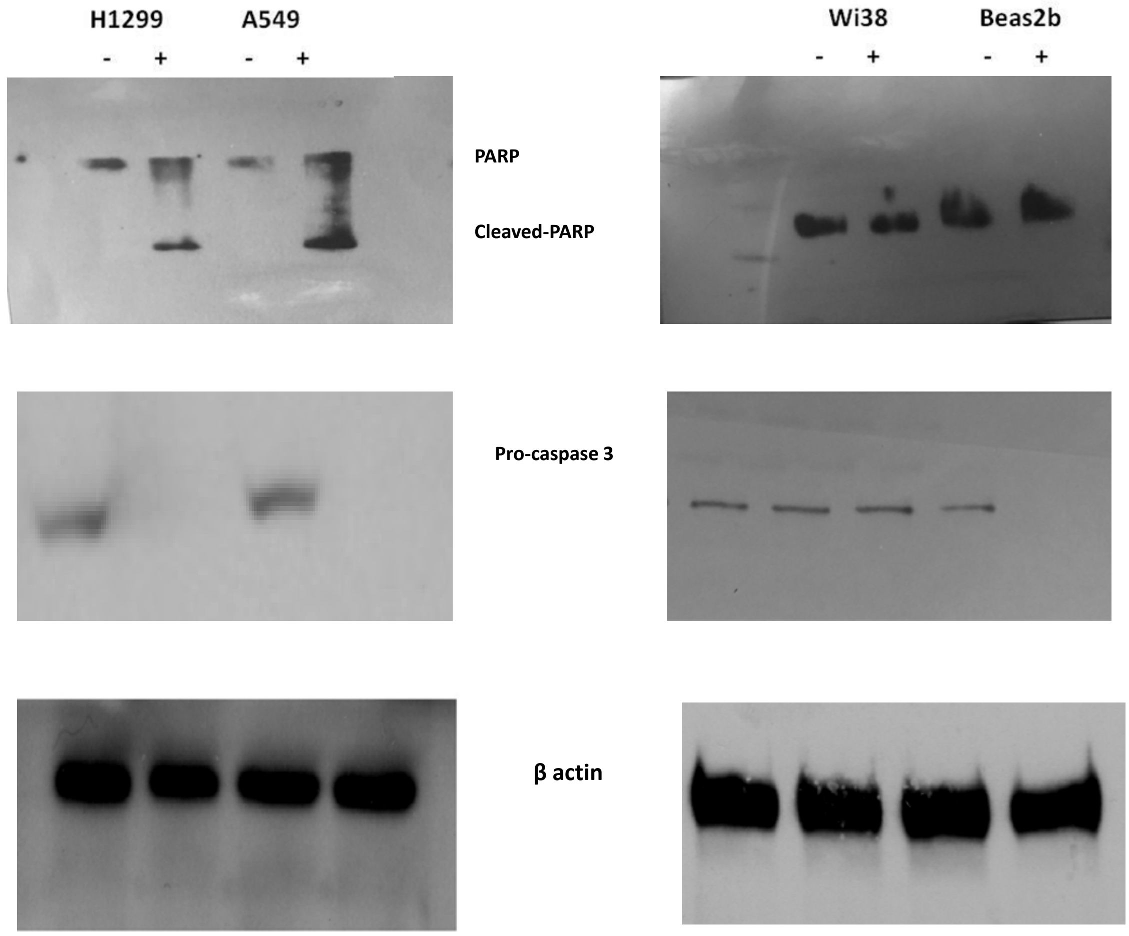

Figure S1. Western Blot analyses of the proteins involved in apoptosis of NSCLC (H1299 and A549) cell lines, normal bronchial epithelial (Beas2b) cells and human fibroblasts (Wi38) . The Western Blot analyses for the indicated proteins were performed on the protein lysates from cells, which were incubated with (+) and without (-) *Urtica dioica* extract (60 μg/mL) for 72 h. The blots have been cut out according to the molecular weigh of the proteins to allow multiple hybridizations with specific antibodies including anti-actin antibody that was used to normalise the obtained signals.

After hybridization, the cropped images were acquired and the blots were re-hybridized where applicable.

Figure S2.

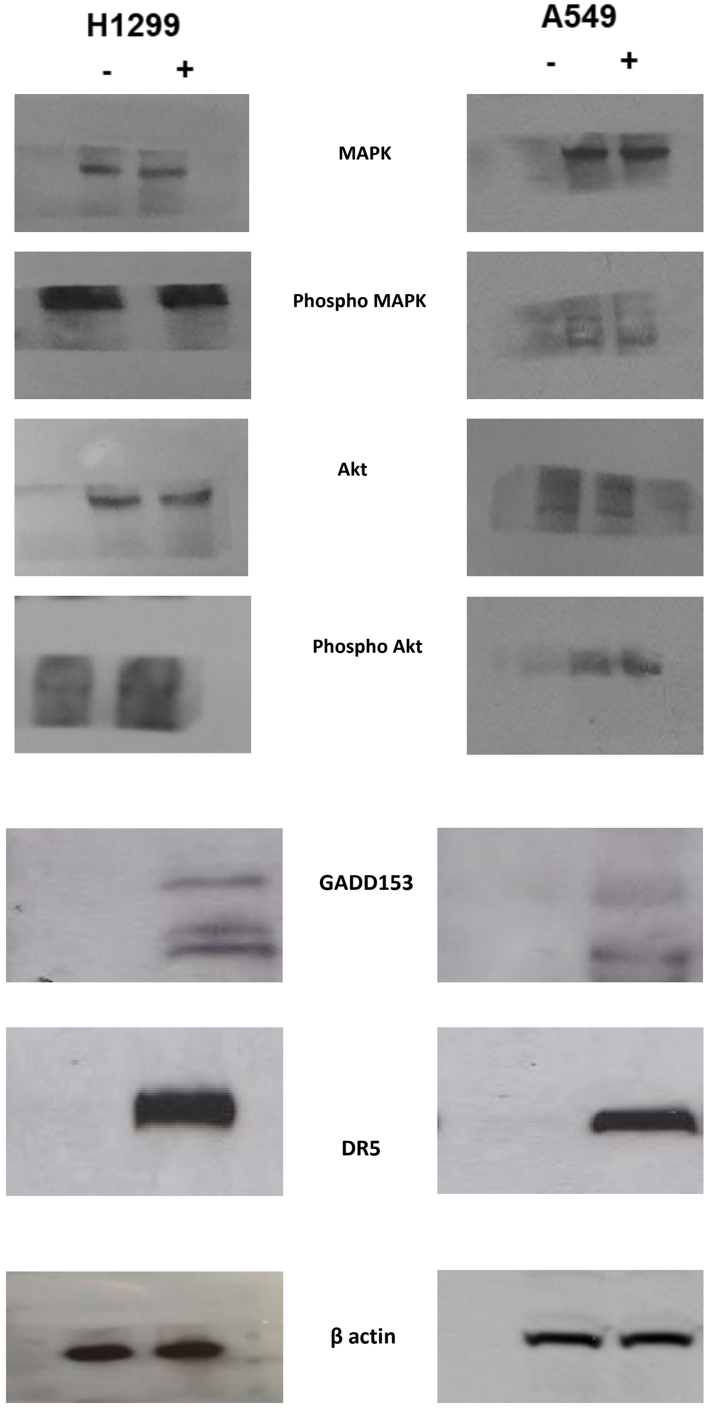

Figure S2. The expression levels of ER-stress related proteins. The expression levels of ER-stress related proteins were investigated on H1299 and A549 cancer cells following treatment with (+) and without (-) *Urtica dioica* extract (60 μg/mL) for 72h. The blots have been cut out according to the molecular weigh of the proteins to allow multiple hybridizations with specific antibodies, including anti-actin antibody that was used to normalize the obtained signals. After hybridization, the cropped images were acquired and the blots were re-hybridized where applicable.

Figure S3A. CIGAR experiment of *Urtica dioica* crude extract. R= rutin. OXY=oxylipins

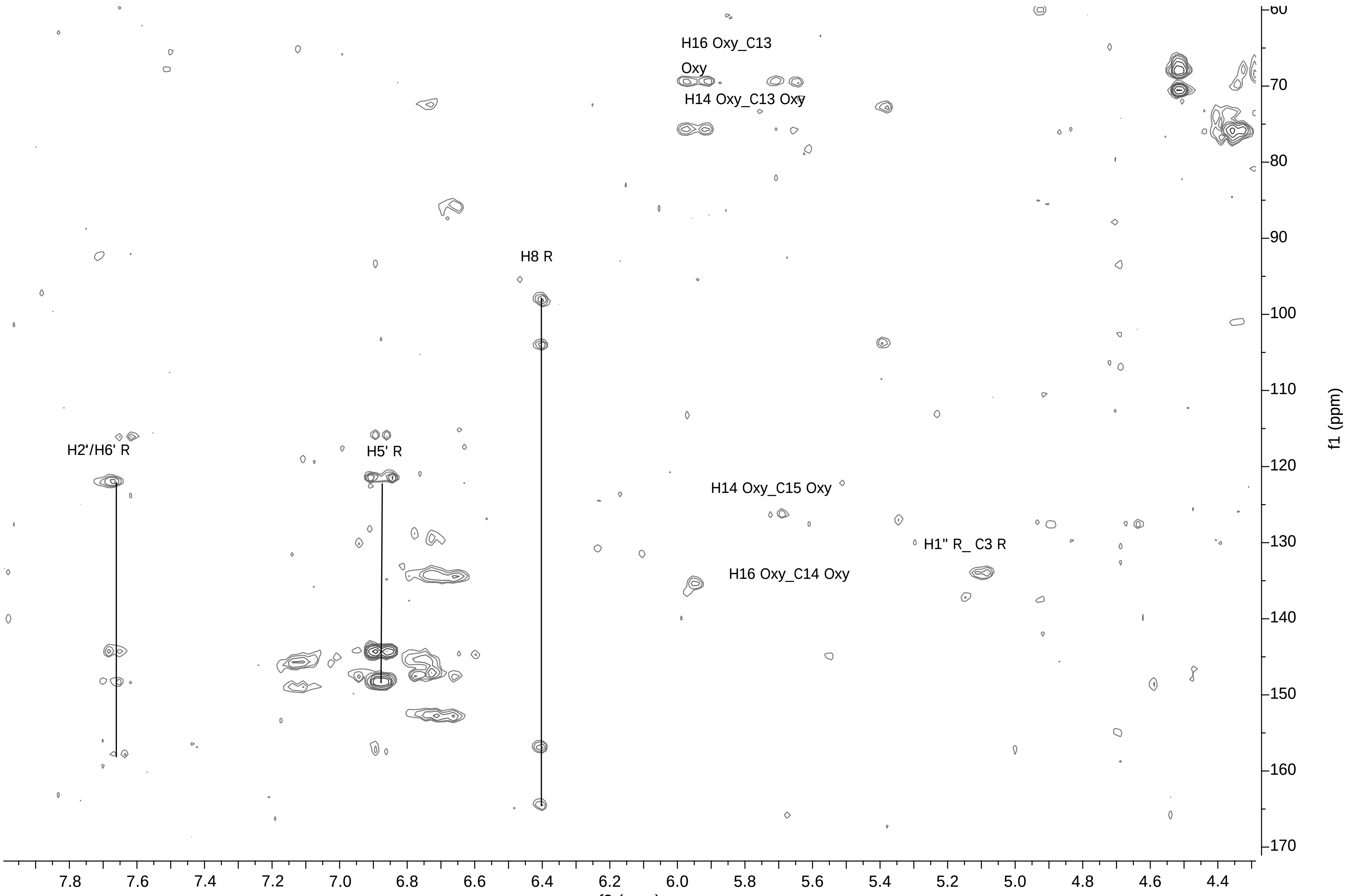

Figure S3B. Aliphatic region of CIGAR experiment of *Urtica dioica* crude extract, R= rutin. OXY=oxylipins

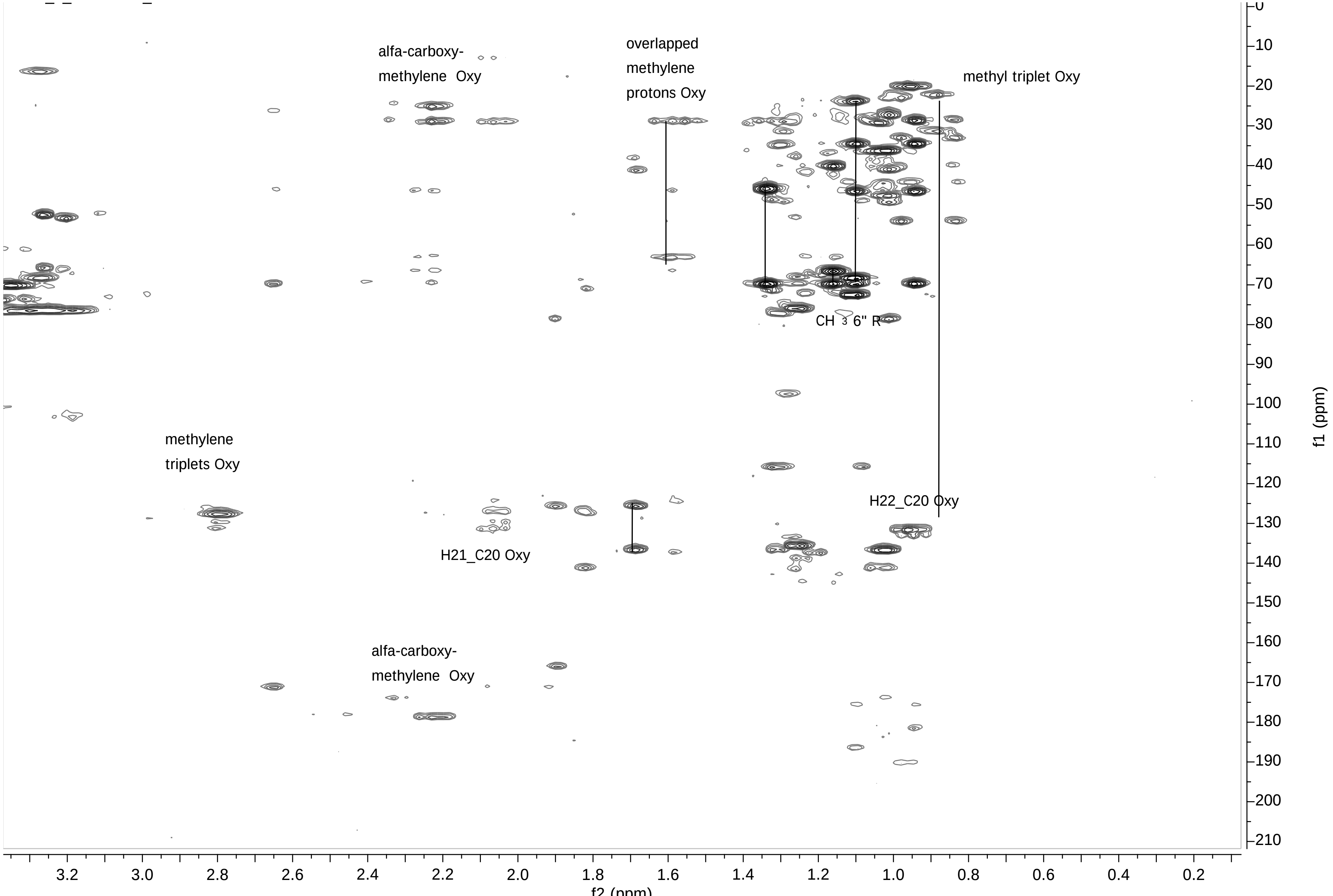

Figure S4. HSQC-TOCSY experiment of *Urtica dioica* crude extract

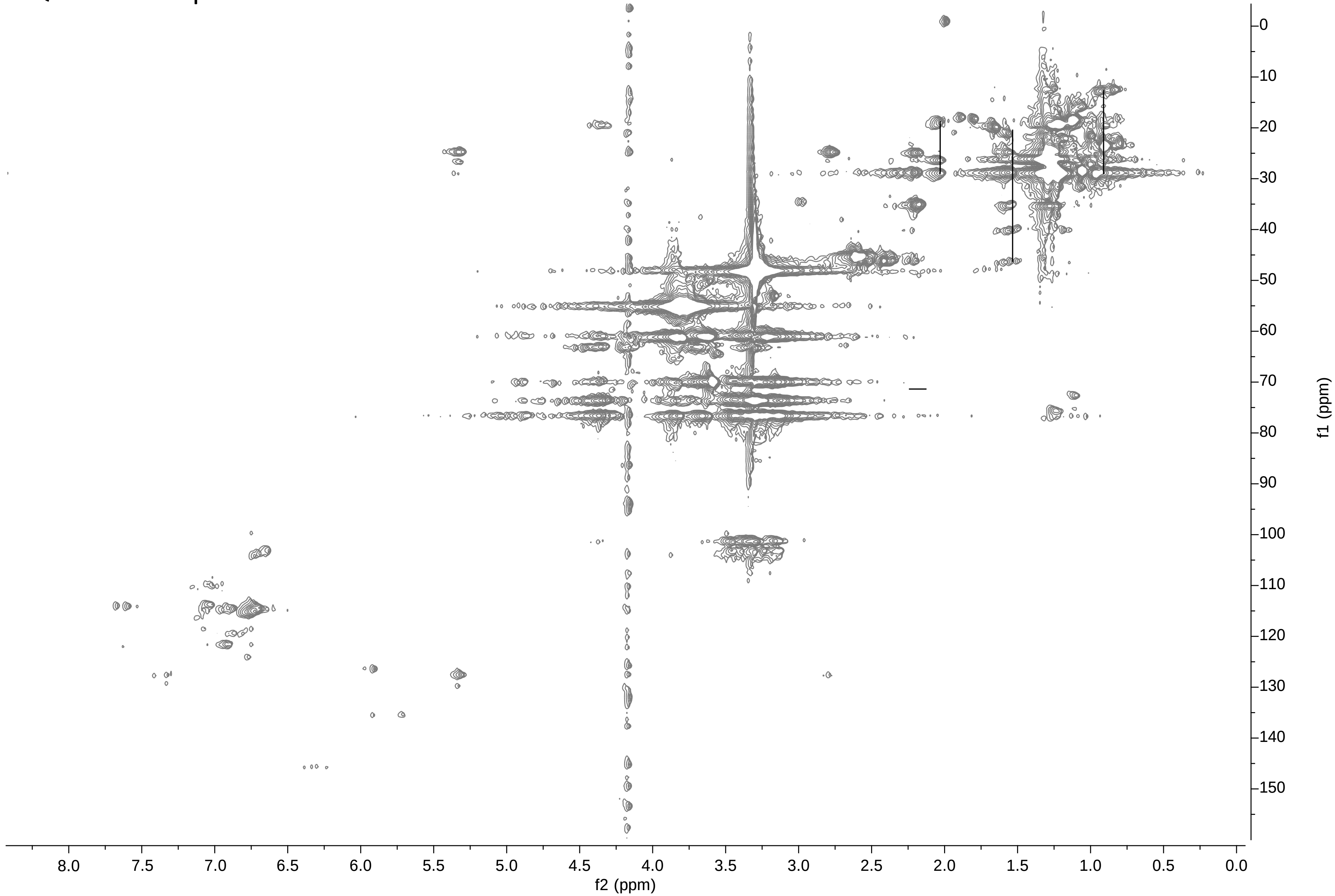

Figure S5. DQCOSY experiment of *Urtica dioica* crude extract. R= rutin. OXY=oxylipins

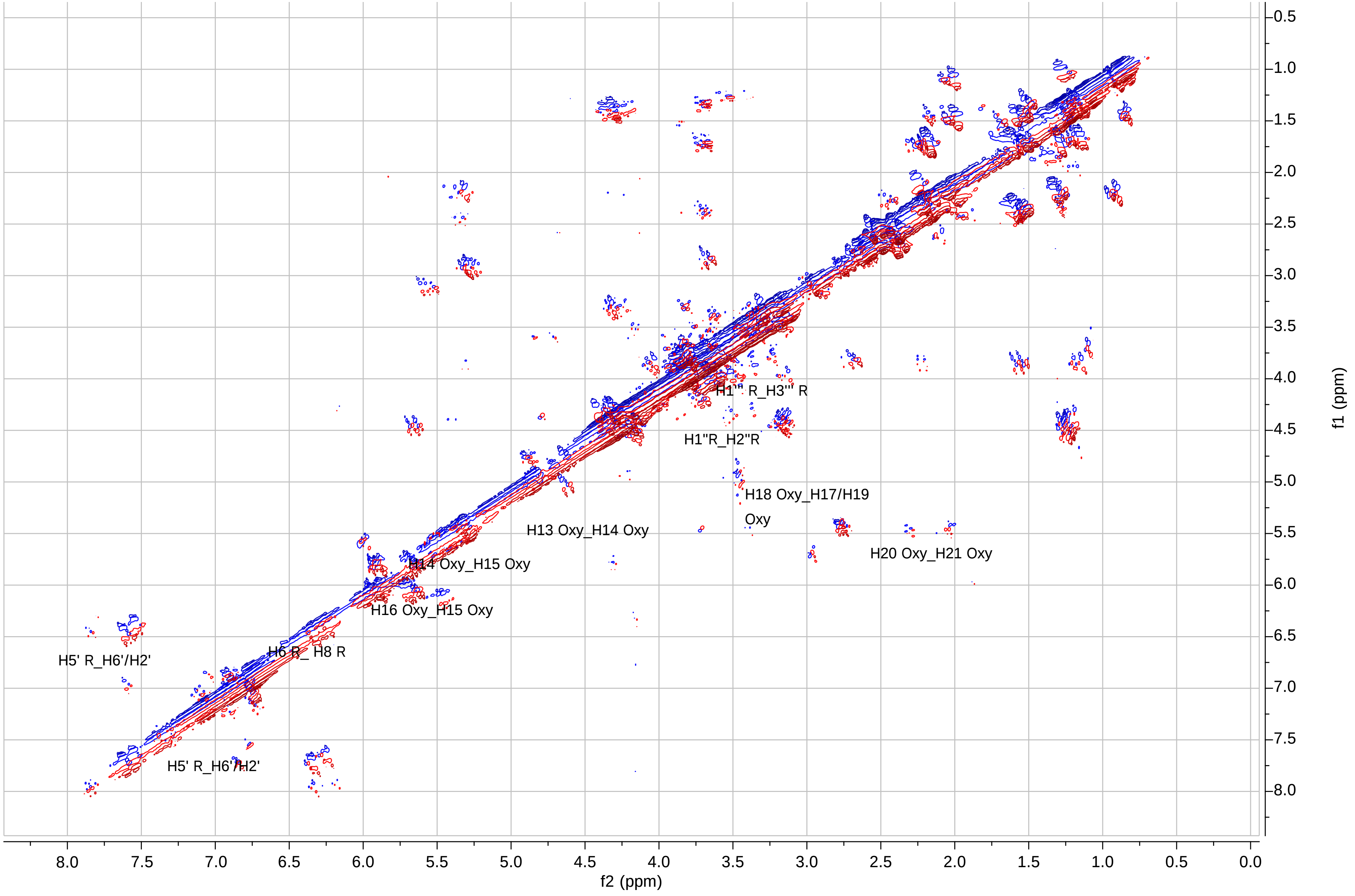

Figure S6. HMBC experiment of rutin

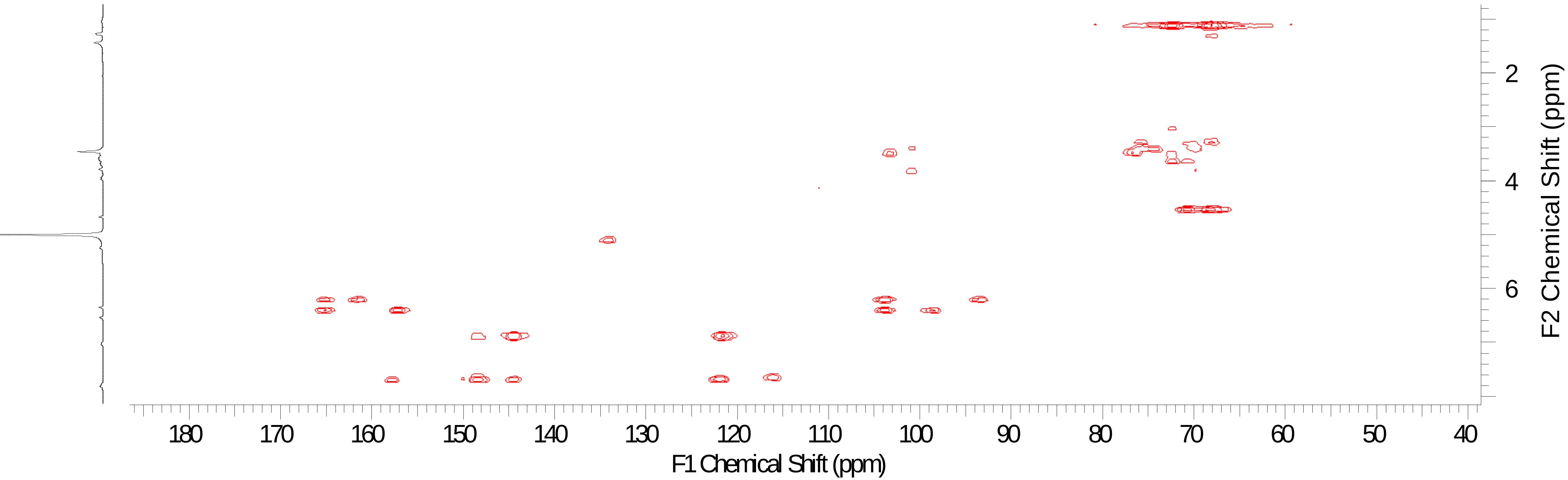

Figure S7. ^{1}H-NMR spectrum of oxylipins' enriched fraction

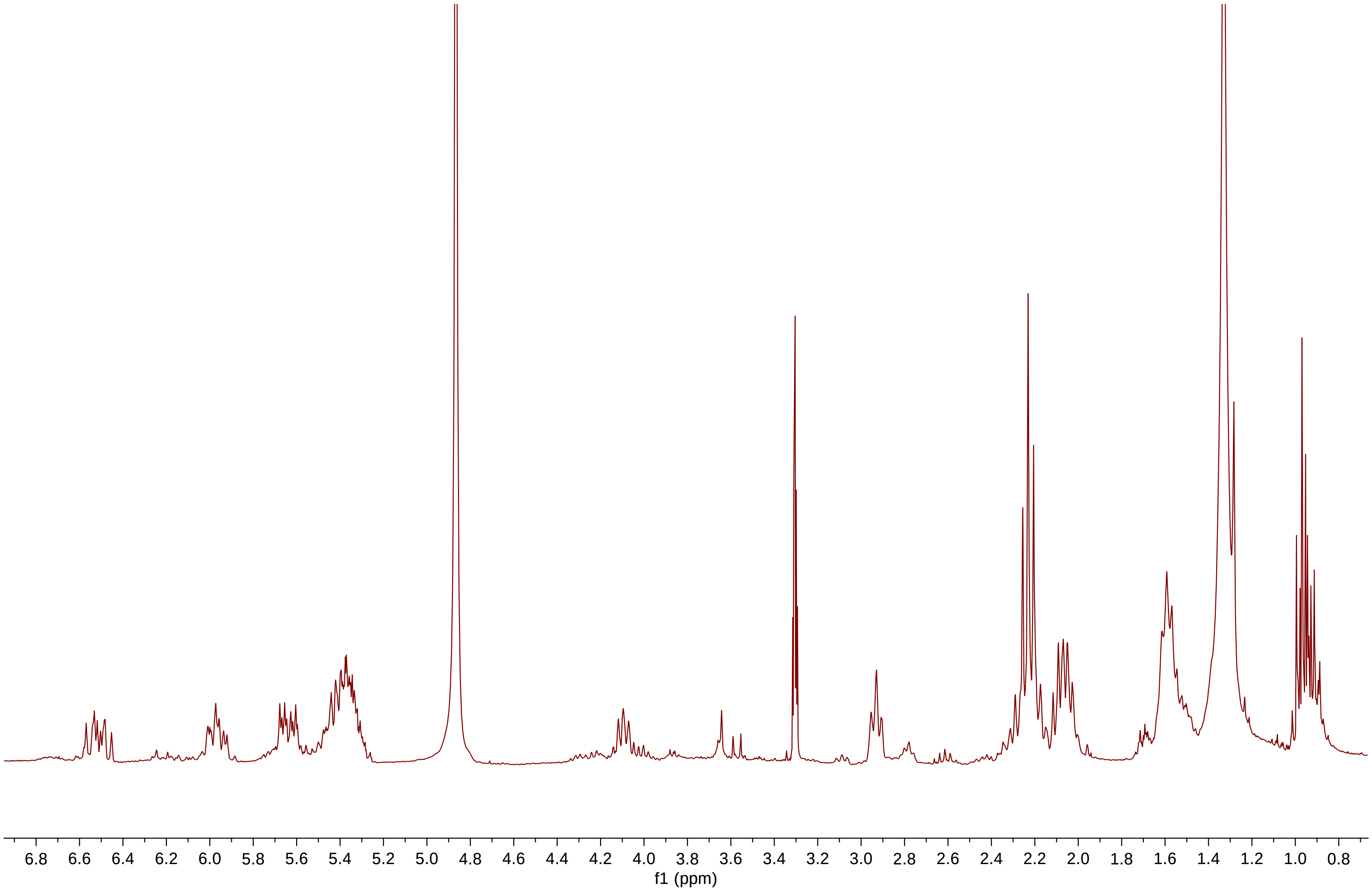

Figure S8. H2BC experiment of oxylipins enriched fraction

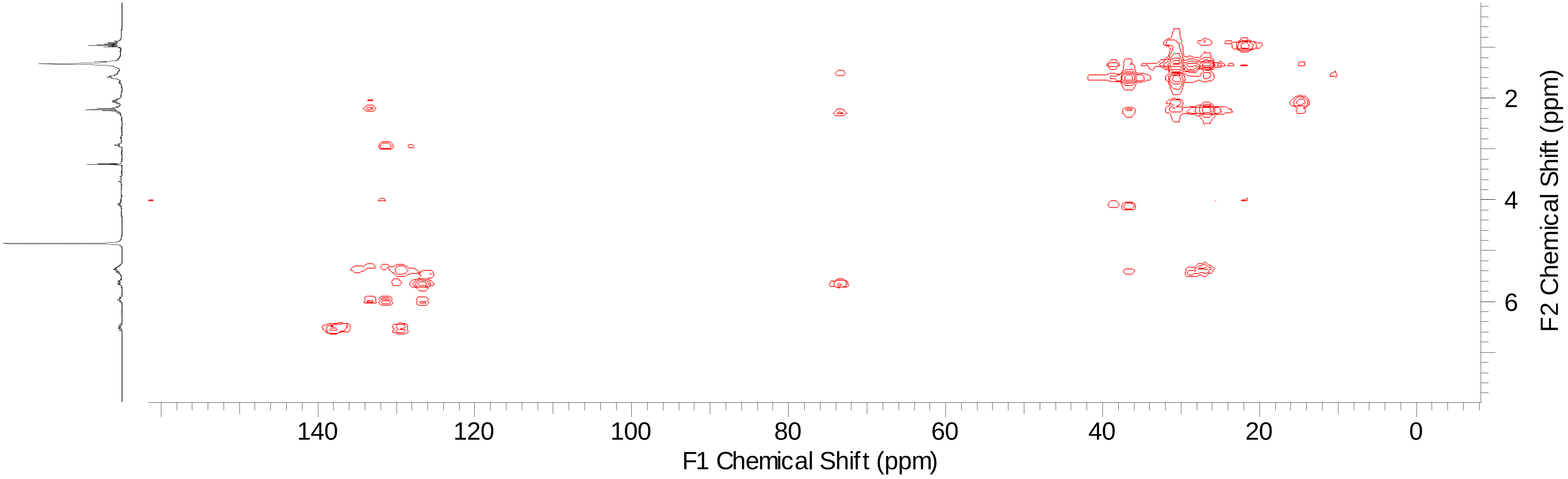

Figure S9. ESI Q-TOF MS SPECTRUM of main oxylipin acquired in negative (A) and positive ion mode (B)

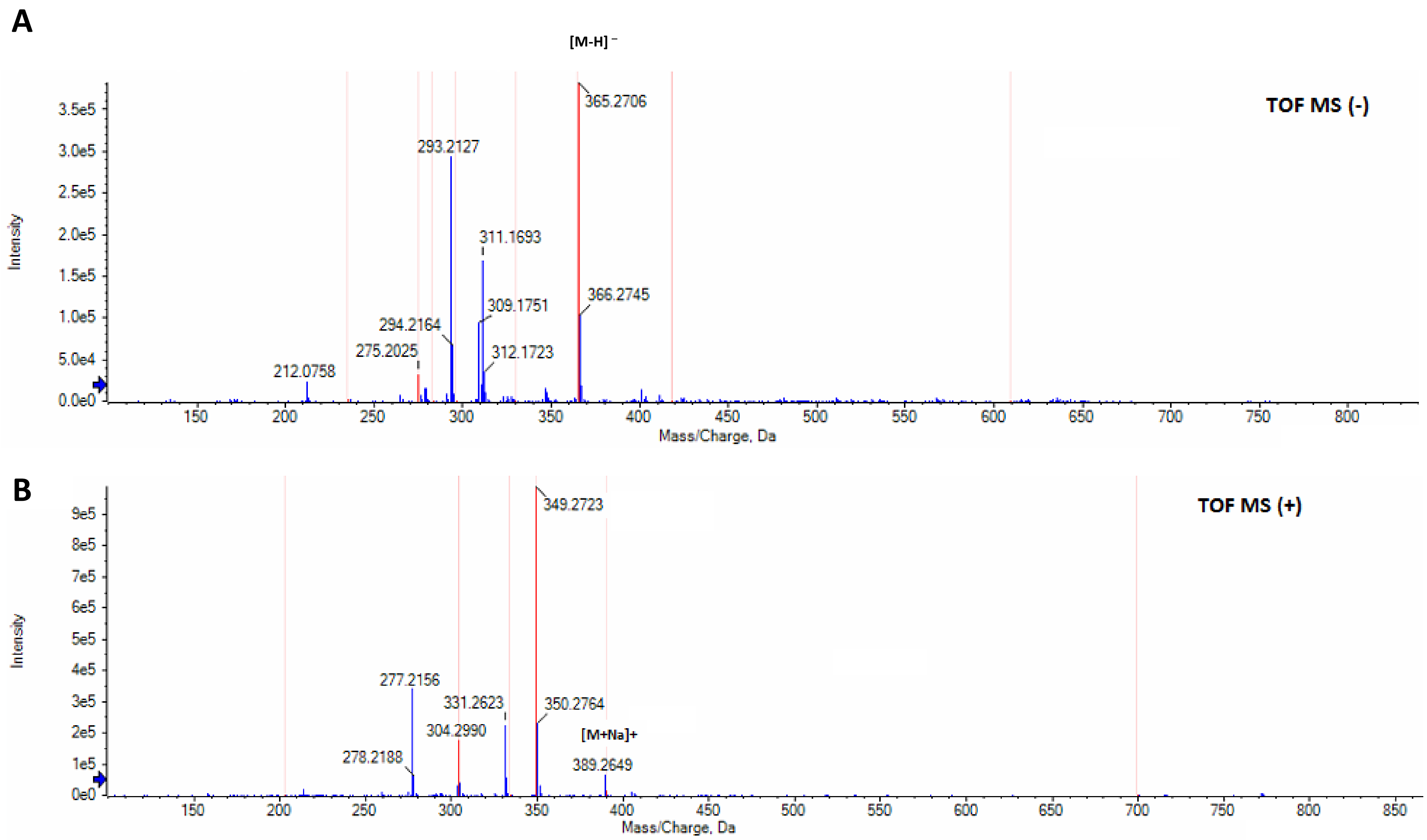

Figure S10 HSQC experiment of the main oxylipin. A aliphatic region. B aromatic region

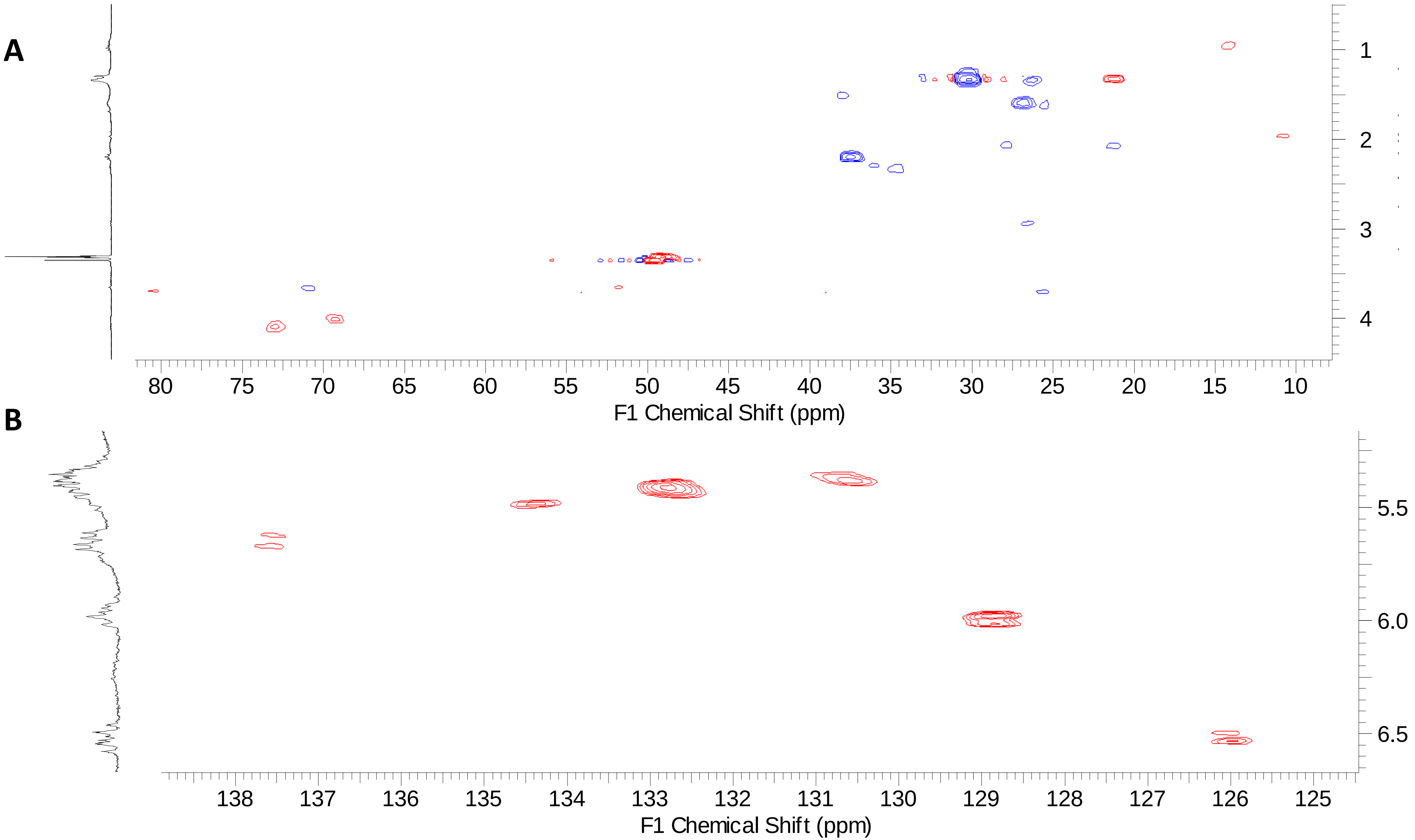

Table S1.NMR data of oxylipin recorded in CD_3OD. δ_H and δ_C are measured in ppm

Position	δ_H ,m (J in Hz)	δ_C , (m)	HMBC (H$\rightarrow$C)
1	-	182.4 (C)	
2	2.21 t (7.2)	37.8 (CH_2)	C1
3	1.61 m	26.8 (CH_2)	C1
4	1.35 ov	30.6 (CH_2)	C1
5	1.35 ov	30.5 (CH_2)	
6	1.35 ov	30.5 (CH_2)	
7	1.35 ov	30.6 (CH_2)	
8	1.61 ov	26.1 (CH_2)	
9	1.51 sv	38.4 (CH_2)	
10	4.00 ov	69.2(CH)	C9, C8
11	2.22 ov	28.3 (CH_2)	
12	2.27 ov	36.1 (CH_2)	
13	4.09 ov	72.9 (CH)	C12, C11, C10
14	5.66 dd (6.6, 15.0 Hz)	137.8 (CH)	C13
15	6.53 dd (15.0 10.8 Hz)	126.1 (CH)	C13, C16, C17
16	5.99 dt (10.8 11.1 Hz)	128.8 (CH)	C14, C15
17	5.35 ov	131.5 (CH)	
18	2.95 t (7.2)	26.6 (CH_2)	C17, C19, C20
19	5.38 ov	130.8 (CH)	
20	5.43 ov	132.7 (CH)	
21	2.07 m	21.4 (CH_2)	
22	0.99 t (7.5)	14.2 (CH_3)	C20, C21

dd=doublet of doublet, m=multiplet, *ov*=overlapped, t=triplet.
